# PATIENT-CLINICIAN RELATIONSHIPS IN HOME HEALTH CARE

**DOI:** 10.1093/geroni/igad104.1723

**Published:** 2023-12-21

**Authors:** Ayomide Bankole, Tyra Girdwood, Dorothy Addo-Mensah, Mark Toles

**Affiliations:** UNC-Chapel Hill, Chapel Hill, North Carolina, United States; Duke University, Durham, North Carolina, United States; University of North Carolina at Chapel Hill, Chapel Hill, North Carolina, United States; UNC-Chapel Hill, Chapel Hill, North Carolina, United States

## Abstract

Patient-clinician relationships are fundamental attributes of high-quality home health care (HHC); yet little is known about patient-clinician relationships in HHC (where 5 million Medicare beneficiaries receive care annually). The objective of the study was to describe perspectives of HHC patients and HHC clinicians about patient-clinician relationships in HHC. We conducted a secondary qualitative analysis of semi-structured interviews (n=34) from a completed qualitative study investigating perspectives of older adult HHC patients (or caregivers as proxy) and their HHC clinicians (17 pairs) on discharge preparedness in a large HHC organization in North-Carolina. A conceptual model of patient-clinician relationships guided content analysis of the interview data. HHC patients identified as White (65%) and black (35%). Most HHC patients reported female sex (53%) and average age was 83 years (range= 69-93). Clinicians were registered nurses and physical therapists. Across the patient-clinician pairs, HHC patients valued relationships with clinicians with shared commonalities and those who provided reciprocal informational exchange and respected their autonomy. HHC clinicians valued relationships in which they felt helpful and respected as a healthcare professional. Relational conflicts arose when there was discordance in expectations of care (e.g., type of HHC services provided). Conflicts also arose when organizational constraints, such as disruptive scheduling, limited HHC visit time, and when limited continuity of care prevented the formation of patient-clinician relationships. Relational conflicts and organizational constraints influence patient-clinician relationships in HHC. Multi-level interventions (targeting modifiable factors at the patient, clinician, and organizational levels) are needed to improve the patient-clinician relationships and the quality of HHC.

